# Derivation of continuum models from discrete models of mechanical forces in cell populations

**DOI:** 10.1007/s00285-021-01697-w

**Published:** 2021-12-08

**Authors:** Per Lötstedt

**Affiliations:** Division of Scientific Computing, Department of Information Technology, Uppsala University, 751 05, Uppsala, Sweden

**Keywords:** Biomechanics, Cell forces, Coarse-graining, Macroscale, Microscale, 92C10, 92C17, 70-08, 65M08

## Abstract

In certain discrete models of populations of biological cells, the mechanical forces between the cells are center based or vertex based on the microscopic level where each cell is individually represented. The cells are circular or spherical in a center based model and polygonal or polyhedral in a vertex based model. On a higher, macroscopic level, the time evolution of the density of the cells is described by partial differential equations (PDEs). We derive relations between the modelling on the micro and macro levels in one, two, and three dimensions by regarding the micro model as a discretization of a PDE for conservation of mass on the macro level. The forces in the micro model correspond on the macro level to a gradient of the pressure scaled by quantities depending on the cell geometry. The two levels of modelling are compared in numerical experiments in one and two dimensions.

## Introduction

Mathematical modelling for simulation of cell populations is a tool complementing experimental studies to understand the complex biochemical and mechanical interactions between the cells in aggregations of unicellular organisms in bacterial cell colonies (Hellweger et al. 2016) and in multicellular organisms forming growing and developing tissues (Liedekerke et al. 2015; Lowengrub et al. 2010).

The mathematical models are either continuum models or discrete models. In continuum models, the evolution of the cell population can be modelled by the solution of time dependent, nonlinear partial differential equations (PDEs) for the cellular densities and concentrations of chemical compounds (Brodland et al. 2006; Frieboes et al. 2010; Humphrey 2003). In contrast, each cell, its internal state, and its motion are followed in time dependent discrete models (Liedekerke et al. 2015). There are techniques for analysis of a macroscale, continuum PDE model, for inference of parameters from experimental data on the population level, and established methods for the numerical solution of it. The continuum model is computationally efficient but it does not resolve any cellular details and heterogeneities in the population. A microscale, discrete model incorporates details of the cell behavior such as local chemical and mechanical interactions between cells, cell proliferation, and cell death. The number of cells can be a few to billions (Byrne and Drasdo 2009; Kang et al. 2014). Large numbers of cells may be necessary to bridge the gap between the individual cell level at the $\mu m$ scale and the population level at the *mm* or *cm* scale. The problem is that the computational effort to simulate billions of individual cells may be prohibitive. By coarse-graining (or upscaling) the microscopic forces to the macroscale, large savings in computing time are possible.

In this paper, we derive novel continuum models from discrete models in one, two, and three dimensions (1D, 2D, 3D) for the biomechanical forces between the cells using the similarities between certain discrete models and discretizations of PDEs by a finite volume method (FVM) and of a pressure gradient. The force terms in the systems of ordinary differential equations (ODEs) governing the discrete models are identified as spatial discretizations of PDEs by the FVM. For a complete cell model we also need models for the motion in response to chemical gradients as in chemotaxis and haptotaxis, equations for the evolution of chemical species internally in the cells e.g. for the gene expression and cell metabolism, and externally e.g. for the signalling between cells, and transport of nutrients and oxygen. These models can also be continuous or discrete but are not addressed here.

Two different kinds of discrete models for the biomechanics of cells can be distinguished: on-lattice and off-lattice models. The cells are constrained in space to a lattice in on-lattice models. Each lattice point in a cellular automata (CA) model can host one or more cells. The cells move by stochastic jumps between the lattice points. A cell occupies several lattice sites in a cellular Potts (CP) model allowing greater geometric flexibility (Liedekerke et al. 2015). The cells move off-lattice and continuously in space and time in an agent based model (ABM) (Liedekerke et al. 2015; Osborne et al. 2017). Each individual cell can be modelled as an agent in an ABM and the motion of the cell satisfies Newton’s equations of motion. The mechanical properties of the cells can be described in more detail in an off-lattice model with separate moving cell entities compared to the on-lattice CA and CP models but the latter are simpler and faster to simulate. Other advantages and disadvantages of ABM compared to CA and CP are discussed in Hellweger et al. (2016) and comparisons are made between CA, CP, and ABM models in Osborne et al. (2017).

A center based model (CBM) and a vertex based model (VBM) are ABMs where each cell is represented individually. The geometry of a cell in a CBM is a circle in 2D or a sphere in 3D in the overlapping spheres (OS) model (Drasdo and Höhme 2005) or a polygonal Voronoi cell defined by Voronoi tesselation (VT) (Kennedy et al. 2016). The forces due to the interactions between neighboring cells and external forces are applied at the cell center in OS and VT models. The adhesion and repulsion forces depend on the distance between the cell centers and parameters in various ways in the models (Ghaffarizadeh et al. 2018; Liedekerke et al. 2019). A summary and an evaluation of different CBM forces are found in Mathias et al. (2020). The cell membrane is modelled by a polygon (2D) or a polyhedron (3D) obtained by Voronoi tesselation in a VBM (Farhadifar et al. 2007; Fletcher et al. 2013, 2014; Honda et al. 2004; Murisic et al. 2015; Nagai and Honda 2001; Staple et al. 2010; Weliky and Oster 1990). A motivation for the method is that the Voronoi polygon or polyhedron resembles the cell shape in certain tissues (Schaller and Meyer-Hermann 2005). The forces are applied at each vertex of the membrane and depend on the cell volumes and the perimeters of the cells adjacent to the vertex. Some vertex models are derived from an energy potential (Farhadifar et al. 2007; Murisic et al. 2015; Staple et al. 2010) which is minimized for the stationary solution. A local minimum of the potential will be reached depending on the initial state. All models have constant parameters and the sensitivity to these parameters in a VBM is investigated in Kursawe et al. (2017). The CBM and VBM are implemented in software such as Biocellion (Kang et al. 2014) , CellSys (Hoehme and Drasdo 2010), Chaste (Cooper et al. 2020; Mirams et al. 2013), MecaGen (Delile et al. 2017), and PhysiCell (Ghaffarizadeh et al. 2018). CP models are implemented in CompuCell3D (Swat et al. 2012) and Morpheus (Starruß et al. 2014).

The motion of the cells in CBM and VBM is overdamped in a viscous environment with small intertial terms compared to the disspative terms (Danuser et al. 2013; Fletcher et al. 2013) and the second derivatives in time in Newton’s equations can be neglected. Thus, the CBM and the VBM are governed by a system of ODEs of first order in time for the coordinates of the cell centers (CBM) or the cell vertices (VBM).

The above deterministic models can be extended in different directions. A stochastic term models the micro-motility of a free cell as Brownian motion in the overview in Earnest et al. (2018) and also in Buttenschön et al. (2018), Middleton et al. (2014). The cells are ellipsoidal in Jin and Marshall (2020) with forces due to adhesion, lubrication, and hair-like appendages and bacterial biofilms are simulated with the model. Problems with growing domains are treated in Baker et al. (2010). Non-local effects between the cells correspond to integro-differential equations on the macro level (Buttenschön et al. 2018; Giniūnaite et al. 2020; Middleton et al. 2014).

The relation between the micro level ABMs and macro level ODEs and PDEs has been studied in An et al. (2017), Byrne and Drasdo (2009), Drasdo (2005), Fozard et al. (2010), Liedekerke et al. (2015), Osborne et al. (2010) aiming at finding a direct correspondence between the models and their parameters at the two levels of modelling. If an expensive micro model is replaced by a cheaper macro model, then computational time and space can be saved. One approach is to first obtain an equation for the probability density of finding a cell at a certain position and then from that derive a PDE for the mean field of the cell density. Another approach is to regard the cell model as a finite difference approximation of a PDE which will be the macro model. The discrete micro level and the continuum macro level have been coupled in a number of papers, mostly in 1D. A nonlinear diffusion PDE in 1D is derived from a spring model for the cell forces in Murray et al. (2009, 2012). The diffusion coefficient is proportional to the spring constant and the viscosity coefficient and inversely quadratically proportional to the cell density in Murray et al. (2009). Another 1D continuum model is derived in Fozard et al. (2010) from an ABM with linear spring forces between the cells and drag due to the ambient substrate. The linear spring parameters in the discrete model in 1D in Murphy et al. (2019) are space dependent. The analysis shows how the parameters on the macro and micro levels are directly related. A mean field PDE in 1D for the motion and proliferation of cells is derived from a stochastic individual-based model in Chaplain et al. (2020). The resulting PDE is a diffusion equation with a source term. The CP model with stochasticity is the micro model in 1D and 2D in Lushnikov et al. (2008). A PDE with a nonlinear diffusion coefficient is derived there for the cellular density. A vertex model for an epithelium is homogenized to arrive at a linear elastic thin plate model in 2D at the PDE level which is valid on long length scales in Murisic et al. (2015). By adding a term in the potential of the vertex model, deformations in 3D are possible. A more elaborate cell geometry than that defined by Voronoi tesselation for a VBM is proposed in Jensen et al. (2020) to obtain a planar stress model at the macro scale.

The tissue level and the cell level are merged in computational hybrid models making them suitable for multiscale simulations of large aggregations of cells. Some examples follow. A PDE model for elasticity on the macro level is combined with a micro model on the cell level in Ghysels et al. (2009). The model for individual cells has spring forces and cell volume preservation. The PDE is discretized by a finite element method and the micro model is used at discrete points in space as in the heterogeneous multiscale method (Weinan et al. 2007). The forces in the stochastic on-lattice method in Engblom et al. (2018) are given by the solution of a PDE. A computational hybrid model of a tumour with individual cells in a proliferating outer layer and a continuum model in the interior of the tumour is proposed in Kim et al. (2007). In this way, expensive cell simulations are avoided in the quiescent center of the tumour.

### Outline of paper

Here we establish relations between the mechanical properties of biological cells modelled by the CBM and the VBM on the discrete micro level and PDEs on the continuum macro level in 1D, 2D (CBM, VBM), and 3D (CBM). A center of cell *i* in a CBM with coordinates ${\mathbf {x}}_i$ on the micro level is advanced in time by the ODE

1 $$\begin{aligned} \frac{\mathrm{d}{{\mathbf {x}}}_i}{\mathrm{d}t}={\mathbf {v}}_i=\sum _{j}{\mathbf {f}}_{ij}, \end{aligned}$$

where ${\mathbf {v}}_i$ is the velocity, ${\mathbf {f}}_{ij}$ is the force between cell *i* and cell *j*, and the sum is over all other cells in the neighborhood of cell *i*. The same type of equation is solved in the VBM for the vertex coordinates. Then the sum in (1) is taken over all other vertices in the neighborhood. The equations for the coordinates of the cell centers and vertices are written in Lagrange coordinates following the flow. The spatial domain with the cells is tiled by Voronoi tesselation with one cell center in each Voronoi element defining the geometry of the cell as in the CBM-VT model.

The PDE for the evolution of the cell density $\rho$ on the macro level is

2 $$\begin{aligned} \frac{\partial \rho }{\partial t}+\nabla \cdot (\rho {\mathbf {v}})=0,\quad {\mathbf {v}}=-\mu \nabla p, \end{aligned}$$

with a relation between the velocity and the pressure *p* according to Darcy’s law for flow in a porous medium. It is written in Euler coordinates fixed in space. For closure of (2), a constitutive relation between *p* and $\rho$ is needed.

The space derivatives in (2) are discretized by a FVM on the Voronoi mesh. There is one cell per interval (1D), area (2D), or volume (3D) element in the discretized PDE and consequently, the cell density $\rho$ is the reciprocal of the element size. By comparing the expressions for the macroscale velocity of the discretization of the PDE in (2) with the microscale velocity in (1) for the CBM, we find that the appropriately scaled pressure agrees with the CBM forces. Similarly for the VBM, the pressure gradient in (2) can be identified with microscale force terms in (1) after scaling. The scaling for both CBM and VBM depends on the cell geometry. Since it is not known in detail at the PDE level in 2D and 3D, some geometrical assumptions are necessary to determine the scaling of the forces between individual cells to obtain the pressure. The main contribution of the paper is the derivation of the relation between the CBM and VBM forces ${\mathbf {f}}_{ij}$ on the micro level in (1) and the pressure *p* in the PDE (2) on the macro level.

When the PDE has been obtained, it can be solved numerically on any mesh, not only on the one defined by the geometry of the cells. If the variation of $\rho$ is small, then a much coarser mesh than the Voronoi mesh for the cells is possible with savings in computational work and memory. In numerical experiments, ABM simulations of distributions of cells of high and low density are compared with PDE solutions on equidistant grids in 1D and Cartesian grids in 2D.

The paper is organized as follows. In the next section, the geometry of the cells and the forces between them are specified. In Sect. 3, macro level pressure is derived from the micro level descriptions by comparison with discretizations by FVM and a pressure gradient on Voronoi meshes. The PDEs are discretized by FVM on an arbitrary mesh in Sect. 4 using the pressure formulas. Section 5 contains the numerical experiments with the discrete and continuum models and conclusions are drawn in the final section.

## Cell geometry and forces

The geometry of a biological cell is determined by the Voronoi tesselation based on the cell centers. The cell is an interval in 1D, a polygon in 2D, and a polyhedron in 3D. The cell centers are connected by the edges of the triangles (2D) or tetrahedra (3D) in the associated Delaunay triangulation of the cell centers. The forces in the CBM act along the Delaunay edges and in the VBM the forces act on the vertices of the polygons along the Voronoi edges. This section is a summary of the notation, the cell geometry, and the CBM and VBM forces. Vectors are written in boldface $\mathbf {A}$ and ${\mathbf {x}}$ and ${\dot{{\mathbf {x}}}}$ denotes a time derivative $\mathrm{d}{\mathbf {x}}/\mathrm{d}t$ of ${\mathbf {x}}$.

### Cell geometry

The total number of cells in the system is *N*. Introduce in *d* dimensions ($d=1,2,3$) the coordinates ${\mathbf {x}}_i=(x_{i1},\ldots ,x_{id})^T,\; i=1,\ldots ,N,$ of the center of the cell *i*. The position of cell center *j* relative to cell center *i* is ${\mathbf {r}}_{ij},\; i,j=1,\ldots ,N,$ the distance between the centers is $r_{ij}$, and the direction between the neighboring centers is $\hat{{\mathbf {r}}}_{ij}$

3 $$\begin{aligned} {\mathbf {r}}_{ij}={\mathbf {x}}_j-{\mathbf {x}}_i,\; r_{ij}=\Vert {\mathbf {r}}_{ij}\Vert _2=\left( \sum _{k=1}^d(x_{jk}-x_{ik})^2\right) ^{1/2},\; \hat{{\mathbf {r}}}_{ij}=r_{ij}^{-1}{\mathbf {r}}_{ij}. \end{aligned}$$

The full coordinate vector of all cells is denoted by ${\mathbf {x}}^T=({\mathbf {x}}_1^T, {\mathbf {x}}_2^T, \ldots ,{\mathbf {x}}_N^T)$. The coordinates of the nodes or vertices $\alpha , \beta ,\ldots$ on the perimeter of the cell are ${\mathbf {x}}_\alpha , {\mathbf {x}}_\beta ,\ldots$ and their relative positions are as in (3)

4 $$\begin{aligned} {\mathbf {r}}_{\alpha \beta }={\mathbf {x}}_\beta -{\mathbf {x}}_\alpha ,\; r_{\alpha \beta }=\Vert {\mathbf {r}}_{\alpha \beta }\Vert _2,\; \hat{{\mathbf {r}}}_{\alpha \beta }=r_{\alpha \beta }^{-1}{\mathbf {r}}_{\alpha \beta }. \end{aligned}$$

**Fig. 1:**
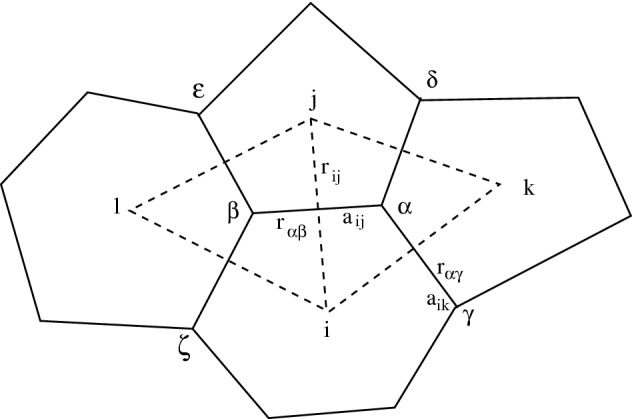
A configuration in 2D of four cells with the primal mesh (solid lines) with cell centers at i,j,k,l, and cell corners at $\alpha ,\beta ,\gamma ,\delta ,\varepsilon ,\zeta$. The centers of two triangles in the dual mesh (dashed lines) are at $\alpha ,\beta ,$ with corners at i,j,k,l

After Voronoi tesselation there are a primal mesh and a dual mesh in Fig. 1. The primal mesh consists of the Voronoi elements representing the biological cells. The Delaunay triangulation defines the dual mesh composed of triangles in 2D. There are four primal elements with centers *i*, *j*, *k*, *l*,  and two dual elements with centers $\alpha , \beta$ in the figure. The vertices $\beta , \gamma , \delta$ are connected to node $\alpha$ in the primal mesh. The three directions $\hat{{\mathbf {r}}}_{\alpha \beta }, \hat{{\mathbf {r}}}_{\alpha \gamma }, \hat{{\mathbf {r}}}_{\alpha \delta }$ start in vertex $\alpha$ and go to vertex $\beta , \gamma ,$ and $\delta$ along edges in the primal mesh. The three edges $\hat{{\mathbf {r}}}_{ij}, \hat{{\mathbf {r}}}_{ik}, \hat{{\mathbf {r}}}_{il}$ in the dual mesh connect the element centers in the primal mesh, see Fig. 1. In an expanded mesh, $\gamma , \delta , \varepsilon ,$ and $\zeta$ will also be triangle centers in the dual mesh.

The interval length in 1D, the element area in 2D, and the element volume in 3D of element *i* are denoted by $V_i$. The perimeter length of an element in 2D is $a_i$ and $a_{ij}$ is the length of the edge separating elements *i* and *j* in 2D. Similarly, the area of the surface between elements *i* and *j* in 3D is denoted by $a_{ij}$ and the perimeter area is $a_i$. In Fig. 1, $a_{ij}=r_{\alpha \beta }$. Since each Voronoi element corresponds to one biological cell, the cell density $\rho$ is $\rho _i=1/V_i$ in element *i* and $V_i$ is the specific volume. Let $\mathcal {J}_i$ be the index set of all adjacent elements to *i*. Then the perimeter $a_i$ of element *i* in 2D and 3D is

5 $$\begin{aligned} a_i=\sum _{j\in \mathcal {J}_i} a_{ij}. \end{aligned}$$

**Fig. 2:**
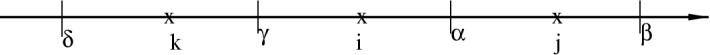
A configuration in 1D with element centers at i,j,k, and boundaries at $\alpha ,\beta ,\gamma ,\delta$

The elements are intervals in 1D. The element indices are *i*, *j*, *k*,  in Fig. 2 and the node indices are $\alpha , \beta , \gamma ,$ and $\delta$. The midpoint of cell *i* is $x_i$ and its length is $V_i=x_\alpha -x_\gamma$.

### Center based models

The cell defined by a Voronoi element in a CBM can be associated with a circle in 2D or a sphere in 3D. Suppose that two circular (2D) or spherical (3D) cells are partly overlapping each other in the OS model. Then there is a force between them repelling the cell centers. There is an attraction force between two cells that are in the vicinity of each other without touching. The force depends on the distance *r* between the cell centers.

The strength of the force is denoted by *g*(*r*). Let *g*(*r*) be defined for $r\ge 0$ with the properties

6 $$\begin{aligned} 0\le r\le s: g(r)\le 0, \quad s<r<r_A: g(r)>0,\quad r> r_A: g(r)=0, \end{aligned}$$

where the parameters $r_A$ and *s* are positive. The cell centers are repelling each other when $r<s$ and attracting each other when $s<r<r_A$. The force vanishes at $r=s$ and for $r>r_A$. The following example of *g* satisfying (6) is from Mirams et al. (2013):

7 $$\begin{aligned} g(r)=\left\{ \begin{array}{cc}\mu (r-s)\exp (-c(r-s)),&{}0\le r\le r_A,\\ 0,&{}r>r_A.\end{array}\right. \end{aligned}$$

There is a discontinuity in *g*(*r*) at $r=r_A$. The parameter $\mu$ determines the strength of the force and $c>0$ the decay rate of the adhesion for $r>s$. Another example is found in Ghaffarizadeh et al. (2018); Macklin et al. (2012):

8 $$\begin{aligned} g(r)=\left\{ \begin{array}{cc} -c_r(1-\frac{r}{r_R})^{n+1}+c_a(1-\frac{r}{r_A})^{n+1},&{} r\le r_R,\\ c_a(1-\frac{r}{r_A})^{n+1},&{} r_R< r \le r_A,\\ 0,&{}r>r_A,\end{array}\right. \end{aligned}$$

This *g*(*r*) is continuous for $r\ge 0$. The adhesion or attraction parameter is $c_a$ and the repulsion parameter is $c_r$. The force model in (8) also satisfies (6) if $c_a<c_r$ and *s* is

$$\begin{aligned} s=\frac{r_Ar_R(c_r^{1/(n+1)}-c_a^{1/(n+1)})}{c_r^{1/(n+1)}r_A-c_a^{1/(n+1)}r_R}<r_R. \end{aligned}$$

In the Hertz model, the repulsion force depends on the cell overlap $r-s$ with $g(r)\sim -(s-r)^{3/2}$ while the adhesion force has a more complicated dependence on $r-s$ (Liedekerke et al. 2015). The Johnson-Kendall-Roberts (JKR) model also depends on $r-s$ and requires the solution of nonlinear equations for *g* (Carpick et al. 1999; Drasdo and Höhme 2005). Other examples of CBM forces are found in Mathias et al. (2020).

The force on cell *i* caused by cell *j* has the magnitude $g(r_{ij})$ and the direction $\hat{{\mathbf {r}}}_{ij}$

9 $$\begin{aligned} \hat{{\mathbf {r}}}_{ij}g(r_{ij}). \end{aligned}$$

Since $\hat{{\mathbf {r}}}_{ij}=-\hat{{\mathbf {r}}}_{ji}$ and $g(r_{ij})=g(r_{ji})$ the forcing on the cell centers *i* and *j* is of equal strength but in opposite directions. Then the system of *d* ODEs for the center coordinates of cell *i* is as in (1) given by Newton’s equations of motion with the viscosity $\eta$, neglecting the accelerations because they are small (Danuser et al. 2013; Fletcher et al. 2013),

10 $$\begin{aligned} {\dot{{\mathbf {x}}}}_i={\mathbf {v}}_i=\frac{1}{\eta }\sum _{j=1, j\ne i}^N\hat{{\mathbf {r}}}_{ij} g(r_{ij}), \end{aligned}$$

and summarized for all cells

11 $$\begin{aligned} {\dot{{\mathbf {x}}}}={\mathbf {F}}({\mathbf {x}}). \end{aligned}$$

The Lagrange coordinates ${\mathbf {x}}$ follow each cell center or how the mass moves in space. This model has the same definition of the force in 1D, 2D, and 3D. There are a limited number of $g(r_{ij})\ne 0$ in (10), see Sect. 3.4. Depending on $r_A$ and the cell size only indices in $\mathcal {J}_i$ may contribute to the sum.

### Vertex based models

The forces are applied in the corners of the cell polygon in 2D in a VBM according to Weliky and Oster (1990). Let the areas of the cells be $\mathbf {V}=(V_1, V_2, \ldots , V_N)^T$ and their perimeters be $\mathbf {a}=(a_1, a_2, \ldots , a_N)^T$. Then the force acting on node $\alpha$ due to node $\beta$ is

12 $$\begin{aligned} \hat{{\mathbf {r}}}_{\alpha \beta }f(\mathbf {V}, \mathbf {a}), \end{aligned}$$

with a direction $\hat{{\mathbf {r}}}_{\alpha \beta }$ and a magnitude $f(\mathbf {V}, \mathbf {a})$ depending on the surrounding $V_i$ and $a_i$. The velocity of node $\alpha$ satifies

13 $$\begin{aligned} {\dot{{\mathbf {x}}}}_\alpha ={\mathbf {v}}_\alpha =\frac{1}{\eta }\sum _{\beta \in \mathcal {J}_\alpha }\hat{{\mathbf {r}}}_{\alpha \beta }f(\mathbf {V}, \mathbf {a}), \end{aligned}$$

where $\mathcal {J}_\alpha =\{\beta ,\gamma ,\delta \}$ in Fig. 1. This equation corresponds to (10) for the CBM. The Lagrange coordinates ${\mathbf {x}}_\alpha$ for the nodes define the shape of the 2D cell. The generalization to 3D and its implementation are more complicated (Honda et al. 2004).

The Weliky-Oster phenomenological force model (Fletcher et al. 2013; Weliky and Oster 1990) at ${\mathbf {x}}_\alpha$ depends on the area and perimeter of cell *k* as follows (see Fig. 1)

14 $$\begin{aligned} \frac{\varsigma }{V_k}\hat{{\mathbf {r}}}_{\alpha \beta }+\kappa a_k(\hat{{\mathbf {r}}}_{\alpha \gamma }+\hat{{\mathbf {r}}}_{\alpha \delta }). \end{aligned}$$

The area force coefficient is $\varsigma$ and $\kappa$ is the perimeter force coefficient. Sum over all three cells with a corner at ${\mathbf {x}}_\alpha$ for the velocity of node $\alpha$

15 $$\begin{aligned} \begin{array}{rl} {\mathbf {v}}_\alpha =&{}\displaystyle {\frac{1}{\eta }\left( (\frac{\varsigma }{V_k}+\kappa (a_i+a_j))\hat{{\mathbf {r}}}_{\alpha \beta }+(\frac{\varsigma }{V_i}+\kappa (a_j+a_k))\hat{{\mathbf {r}}}_{\alpha \gamma }\right. }\\ &{}\displaystyle {\left. +(\frac{\varsigma }{V_j}+\kappa (a_k+a_i))\hat{{\mathbf {r}}}_{\alpha \delta }\right) .} \end{array} \end{aligned}$$

With the force function

16 $$\begin{aligned} f(V, a, b)=\frac{\varsigma }{V}+\kappa (a+b) \end{aligned}$$

in (13), the velocity is

17 $$\begin{aligned} \begin{array}{rl} {\mathbf {v}}_\alpha =&\displaystyle {\frac{1}{\eta }\left( f(V_k, a_i, a_j)\hat{{\mathbf {r}}}_{\alpha \beta }+f(V_i, a_j, a_k)\hat{{\mathbf {r}}}_{\alpha \gamma }+f(V_j, a_k, a_i)\hat{{\mathbf {r}}}_{\alpha \delta }\right) .} \end{array} \end{aligned}$$

Another example is the Nagai-Honda model (Fletcher et al. 2013, App. A), (Nagai and Honda 2001), which is derived by taking the gradient of an energy function. The force at ${\mathbf {x}}_\alpha$ due to $V_k$ and $a_k$ is almost a linearization of (14)

18 $$\begin{aligned} -\lambda (V_k-V_{0k})r_{\alpha \beta }\hat{{\mathbf {r}}}_{\alpha \beta }+(2\beta (a_k-a_{0k})+\gamma _k)(\hat{{\mathbf {r}}}_{\alpha \gamma }+\hat{{\mathbf {r}}}_{\alpha \delta }). \end{aligned}$$

Compared to Nagai and Honda (2001), the force in (18) is slightly simplified here in the factor $r_{\alpha \beta }$ in the first term. The parameter $\lambda$ is a deformation energy coefficient, $\beta$ is a membrane surface energy coefficient, and $\gamma$ is a cell-cell adhesion energy coefficient. The ideal area is $V_{0k}$ and the ideal perimeter length is $a_{0k}$. They are reached in an equilibrium configuration when the forces vanish.

As in Nagai and Honda (2001), the forces in the VBMs in Farhadifar et al. (2007), Murisic et al. (2015), Staple et al. (2010) resemble the force in (18) and are derived from a potential.

Consider a small perturbation from equilibrium in cell *k* (or *i* or *j*) in (14) and (18). The perturbations in ${\mathbf {v}}_\alpha$ are equal with both models if

19 $$\begin{aligned} \lambda r_{\alpha \beta }=\varsigma /V_{0k}^2,\; \kappa =2\beta ,\; \gamma _k=0. \end{aligned}$$

## Comparison of the microscale models with a macroscale PDE

A macroscale PDE is derived for the cell density $\rho$ in this section. The density is transported by a pressure gradient. The PDE is discretized by a FVM on the Voronoi mesh defining the biological cells. The discretized pressure gradient on the macro level corresponds to the forces in the CBM and the VBM on the micro level. The geometrical detail of the cells used in the CBM and the VBM on the microscale is missing on the macroscale. The cell size is available as $1/\rho$ but, for example, the cell perimeter is not known. By assuming a regular shape of the cell, the size of the edges and the distance between the center and the vertices can be obtained as functions of the cell size. The pressure will then be a function of $\rho$ determined by the specific CBM or VBM.

The viscosity coefficient in (10) and (13) is assumed to be $\eta =1$ and is ignored in the formulas. It can always be removed by including it in the force parameters.

### The conservation law

A fluid parcel has volume $\mathcal {V}$ and surface $\mathcal {S}$. The initial position at $t=0$ is at ${\mathbf {x}}_0$ and its velocity field is ${\mathbf {v}}$. Lagrange coordinates ${\mathbf {x}}(t)$ follow the parcel. This parcel is small on the macroscale and will later be identified as the specific volume *V* of a cell. Use Gauss’s formula to arrive at a geometric conservation law for the time evolution of $\mathcal {V}$ at ${\mathbf {x}}({\mathbf {x}}_0, t)$ when $t>0$

20 $$\begin{aligned} {\dot{\mathcal {V}}}=\frac{\mathrm{d}}{\mathrm{d}t}\int _{\mathcal {V}} \mathrm{d}V=\int _{\mathcal {S}} {\mathbf {n}}\cdot {\mathbf {v}}\, \mathrm{d}S=\int _{\mathcal {V}} \nabla \cdot {\mathbf {v}}\, \mathrm{d}V. \end{aligned}$$

In Euler coordinates with fixed ${\mathbf {x}}$ in space we have with the total derivative *D*/*Dt*

21 $$\begin{aligned} \frac{D\mathcal {V}}{Dt}=\frac{\partial \mathcal {V}}{\partial t}+{\mathbf {v}}\cdot \nabla \mathcal {V}=\int _{\mathcal {V}} \nabla \cdot {\mathbf {v}}\, \mathrm{d}V=\mathcal {V}\nabla \cdot {\mathbf {v}}. \end{aligned}$$

Introduce the density $\rho =1/\mathcal {V}$ in (21) to obtain a PDE for conservation of mass in a domain $\varOmega$

22 $$\begin{aligned} \frac{\partial \rho }{\partial t}+\nabla \cdot (\rho {\mathbf {v}})=0. \end{aligned}$$

On the boundary of $\varOmega$ with normal ${\mathbf {n}}$, the boundary condition is ${\mathbf {n}}\cdot \nabla \rho =0$. Then there is no flux of mass across the boundary and the total mass $\int _\varOmega \rho \, \mathrm{d}V$ is constant in time. A 1D model in Fozard et al. (2010) adds an extra term in (22) for internal viscosity in the cells resulting in a linear spring model for the force.

The PDE (20) is discretized in space by a FVM for cell *i* with volume $V_i$ and center at ${\mathbf {x}}_i$ in the Voronoi mesh defining the cell geometries, see Fig. 1. The time derivative is approximated by the Euler forward method at discrete time points $t^n,\, n=1,2,\ldots ,$ starting at $t^0=0$ with timestep $\varDelta t=t^{n+1}-t^n$. The equation for $V_i$ at $t=t^{n+1}$ is

23 $$\begin{aligned} V^{n+1}_i=V^n_i+\varDelta t\sum _{j\in \mathcal {J}_i} {\mathbf {n}}_{ij}\cdot {\mathbf {v}}_{ij}^na_{ij}. \end{aligned}$$

The sum is taken over $\mathcal {J}_i$, the set of all indices of cells sharing a common boundary with cell *i* (at least *j*, *k*, *l* in Fig. 1). The outward normal on the edge (2D) or surface (3D) between cells *i* and *j* is ${\mathbf {n}}_{ij}$ and ${\mathbf {v}}_{ij}$ is also evaluated on that edge or surface.

For the conservation law (22), we consider any fixed Euler mesh with element (or computational cell) area or volume $\omega _i$. The mesh here is fixed in space contrary to the Voronoi mesh for the biological cells which is continuously deformed in time. The elements *i* and $j\in \mathcal {J}_i$ have a common perimeter of size $\sigma _{ij}$ with an outward normal ${\mathbf {n}}_{ij}$ from cell *i* to *j*. The time derivative is approximated by the Euler forward method at discrete time points $t^n,\, n=1,2,\ldots ,$ starting at $t^0=0$ with timestep $\varDelta t=t^{n+1}-t^n$. With FVM discretization in space, the equation for $\rho$ at $t^{n+1}$ is

24 $$\begin{aligned} \rho ^{n+1}_i=\rho ^n_i-\frac{\varDelta t}{\omega _i} \sum _{j\in \mathcal {J}_i} \frac{1}{2}(\rho _i^n+\rho _j^n){\mathbf {n}}_{ij}\cdot {\mathbf {v}}_{ij}^n \sigma _{ij}, \end{aligned}$$

where ${\mathbf {v}}_{ij}$ is evaluated on the edge or surface $\sigma _{ij}$. The sum is taken over $\mathcal {J}_i$, the set of all indices of elements sharing a common boundary with element *i*. The density at $\sigma _{ij}$ is approxmated by $0.5(\rho _i+\rho _j)$.

Now there are two alternatives: compute the cell density with the continuous model (24) *or* first advance the cell centers with the discrete model (10) or (13) and then compute $V_i$ using (23) or the present geometry of the cell and $\rho _i$ for each cell *i*. If the elements can be chosen much larger than the cells, $\omega _i\gg V_i$, then the first alternative will save computing time and memory. This is possible if $\rho$ varies slowly in space.

We need a relation between ${\mathbf {v}}$ and $\rho$ for closure of (22). In Darcy’s law, ${\mathbf {v}}$ is proportional to the gradient of a pressure *p*

25 $$\begin{aligned} {\mathbf {v}}=-\mu \nabla p. \end{aligned}$$

Inserted into (22) the PDE is

26 $$\begin{aligned} \frac{\partial \rho }{\partial t}=\mu \nabla \cdot (\rho \nabla p). \end{aligned}$$

The equation is closed with a constitutive relation between *p* and $\rho$: $p=p(\rho )$. This is the macroscopic PDE for the evolution of $\rho$ in Chaplain et al. (2020) where a source term for injection of mass on the right hand side models cell proliferation on the macro level (see also Byrne and Drasdo (2009)). One cell splits into two in cell division at the micro level thus doubling the cell density gradually or instantaneously and very locally in space. Then the numerical solution of (22) has to resolve a density peak in space and time requiring refinements in the temporal and spatial discretization. If the proliferation occurs frequently in the domain, then an averaged, smooth source term is preferred to model cell division.

The pressure *p* expressed in *V* or $\rho$ will be determined by the CBM and the VBM in the next two sections.

### Center based models

Apply FVM to (25) in a cell *i*. Then the discretized law is

27 $$\begin{aligned} {\mathbf {v}}_i=-\frac{\mu }{V_i}\sum _{j\in \mathcal {J}_i}p_{ij}{\mathbf {n}}_{ij}a_{ij}, \end{aligned}$$

where $p_{ij}$ is computed at the midpoint of the interface between cell *i* and *j*. With the cell indices in the neighborhood of cell *i* in $\mathcal {J}_i$, the equality (10) for the CBM is rewritten in

28 $$\begin{aligned} {\mathbf {v}}_i=\sum _{j\in \mathcal {J}_i}\hat{{\mathbf {r}}}_{ij}g(r_{ij}). \end{aligned}$$

A *g* with the properties (6) is nonzero for a limited number of ${j\in \mathcal {J}_i}$. Since ${\mathbf {n}}_{ij}=\hat{{\mathbf {r}}}_{ij}$, the same velocity is obtained in (27) and (28) if $p_{ij}$ is chosen as

29 $$\begin{aligned} p_{ij}=-\frac{V_i}{\mu a_{ij}}g(r_{ij}). \end{aligned}$$

This is the bridge between the macro and the micro levels but geometric relations between $V_i, a_{ij},$ and $r_{ij}$ are missing to obtain a macroscale pressure *p*(*V*).

### Vertex based models

The relation (25) is discretized in 2D on the dual triangular mesh, see Fig. 1. The triangle with center at ${\mathbf {x}}_\alpha$ has three edges between triangle $\alpha$ and three neighbors $\beta ,\gamma ,$ and $\delta$. The normals of the edges are ${\mathbf {n}}_{\alpha \beta }, {\mathbf {n}}_{\alpha \gamma },$ and ${\mathbf {n}}_{\alpha \delta }$ and are equal to $\hat{{\mathbf {r}}}_{\alpha \beta }, \hat{{\mathbf {r}}}_{\alpha \gamma },$ and $\hat{{\mathbf {r}}}_{\alpha \delta }$. By (25), the pressure and the velocity at the midpoint of the edge between triangle $\alpha$ and $\beta$ are assumed to satisfy

30 $$\begin{aligned} -\mu {\mathbf {n}}_{\alpha \beta }\cdot \nabla p={\mathbf {n}}_{\alpha \beta }\cdot {\mathbf {v}}={\mathbf {n}}_{\alpha \beta }\cdot ({\mathbf {F}}_\alpha +{\mathbf {F}}_\beta ), \end{aligned}$$

where ${\mathbf {F}}_\alpha$ and ${\mathbf {F}}_\beta$ are the VBM forces at ${\mathbf {x}}_\alpha$ and ${\mathbf {x}}_\beta$ in (15). After discretization of (30), the relation between the forces and the pressures $p_\alpha$ and $p_\beta$ at ${\mathbf {x}}_\alpha$ and ${\mathbf {x}}_\beta$ using (17) is

31 $$\begin{aligned} \begin{array}{rcl} \displaystyle {-\mu \frac{p_\beta -p_\alpha }{r_{\alpha \beta }}}&{}\approx &{}{\mathbf {n}}_{\alpha \beta }\cdot ({\mathbf {F}}_\alpha +{\mathbf {F}}_\beta )\\ &{}=&{}\hat{{\mathbf {r}}}_{\alpha \beta }\cdot (f(V_k, a_i, a_j)\hat{{\mathbf {r}}}_{\alpha \beta }+f(V_i, a_j, a_k)\hat{{\mathbf {r}}}_{\alpha \gamma }+f(V_j, a_k, a_i)\hat{{\mathbf {r}}}_{\alpha \delta })\\ &{}&{}+\hat{{\mathbf {r}}}_{\alpha \beta }\cdot (f(V_l, a_i, a_j)\hat{{\mathbf {r}}}_{\beta \alpha }+f(V_i, a_j, a_l)\hat{{\mathbf {r}}}_{\beta \varepsilon }+f(V_j, a_l, a_i)\hat{{\mathbf {r}}}_{\beta \zeta })\\ &{}=&{}f(V_k, a_i, a_j)-f(V_l, a_i, a_j)\\ &{}&{}+f(V_i, a_j, a_k)\hat{{\mathbf {r}}}_{\alpha \beta }\cdot \hat{{\mathbf {r}}}_{\alpha \gamma }+f(V_j, a_k, a_i)\hat{{\mathbf {r}}}_{\alpha \beta }\cdot \hat{{\mathbf {r}}}_{\alpha \delta }\\ &{}&{}+f(V_i, a_j, a_l)\hat{{\mathbf {r}}}_{\alpha \beta }\cdot \hat{{\mathbf {r}}}_{\beta \varepsilon }+f(V_j, a_l, a_i)\hat{{\mathbf {r}}}_{\alpha \beta }\cdot \hat{{\mathbf {r}}}_{\beta \zeta }. \end{array}\nonumber \\ \end{aligned}$$

The pressures $p_\alpha$ and $p_\beta$ are functions of the microscopic forces in (31) and the inner products involving $\hat{{\mathbf {r}}}$, $V_i,$ and $a_i$. They depend on properties of the geometry of the cell polygons.

### Geometry of the cells

We need relations for the cells in (29) and (31) between their area or volume *V*, their perimeter *a*, a distance *r*, and the inner products such as $\hat{{\mathbf {r}}}_{\alpha \beta }\cdot \hat{{\mathbf {r}}}_{\alpha \gamma }$ to be able to find the macroscopic pressure by coarse-graining the microscopic forces. This is easy in 1D but in 2D and 3D complete geometric information is not available and assumptions on the cell geometry are necessary.

#### One dimension

Let $r_i$ be the distance between the center of cell *i* and the cell boundary in 1D in Fig. 2. Then the length of the cell is

32 $$\begin{aligned} V_i=x_\alpha -x_\gamma =2r_i. \end{aligned}$$

There is no perimeter but let $a_{ij}$ in (5) be 1 and $J=2$. The geometric relations between $V_i, a_{ij},$ and $r_{ij}$ in (29) are

33 $$\begin{aligned} r_i=\frac{1}{2} V_i=\xi _1V_i,\; r_{ij}=\frac{1}{2}(V_i+V_j),\; \frac{V_i}{a_{ij}}=V_i,\; a_i=\sum _{j=1}^2 a_{ij}=2. \end{aligned}$$

The inner products are either $-1$ or 1.

#### Two and three dimensions

The PDE approximation of the forces is expected to be the most accurate when there is a smooth and small variation of the cell density. By assuming that the cell shapes are close to regular polygons in 2D and regular polyhedra in 3D, we can derive approximate expressions for the dependence on *V* of $r_{ij}$ and $a_{ij}$ in (29) and $a_i, r_{\alpha \beta },$ and the inner products in (31). The shortest distance between the cell center and the perimeter in cell *i* is denoted by $r_i$ which is equal to the radius of the inscribed circle. Hence, $r_{ij}=r_i+r_j$.

A regular polygon in 2D with *J* corners and *J* edges fulfills

34 $$\begin{aligned} V_i=Jr_i^2\tan (\pi /J),\; a_{ij}=2r_i\tan (\pi /J),\; a_i=\sum _ja_{ij}=2r_iJ\tan (\pi /J). \end{aligned}$$

Introduce $\xi _2(J)=1/\sqrt{J\tan (\pi /J)}$. Then by (34) we find that

35 $$\begin{aligned} r_i=\xi _2(J)\sqrt{V_i},\; \frac{V_i}{a_{ij}}=\frac{1}{2}J\xi _2(J)\sqrt{V_i},\; a_i=\frac{2}{\xi _2(J)}\sqrt{V_i}. \end{aligned}$$

The polygon becomes a circle when $J\rightarrow \infty$ and $\xi _2\rightarrow 1/\sqrt{\pi }$. A triangle yields the lower bound on $\xi _2$. The $\xi _2$-values for different *J* are found in Table 1.

**Table 1:** The factor $\xi _2$ in the relation $r=\xi _2(J) V^{1/2}$ in regular polygons

J	2D name	$\xi _2$
3	Triangle	$(3\tan (\pi /3))^{-1/2}= 0.4387$
4	Square	$(4\tan (\pi /4))^{-1/2}=0.5000$
5	Pentagon	$(5\tan (\pi /5))^{-1/2}= 0.5247$
6	Hexagon	$(6\tan (\pi /6))^{-1/2}= 0.5373$
$\infty$	Circle	$\frac{1}{\sqrt{\pi }}= 0.5642$

The maximum number of inscribed circles of equal radius in other cells touching circle *i* is 6 (the kissing number (Conway and Sloane 1993)). In a densly packed cell colony, *J* will be close to 6. Choosing 5 or 6 for *J* in $\xi _2$ does not alter its value very much in the table and $\xi _2$ appears to vary slowly when $J\rightarrow \infty$. The inner products between $\hat{{\mathbf {r}}}_{\alpha \beta }$ and other $\hat{{\mathbf {r}}}$-vectors in (31) are equal to $\pm \cos (2\pi /J)$ if the cell is a regular polygon with *J* edges.

The growth of epithelia are simulated in Staple et al. (2010) with a planar vertex model. The statistics of the cell geometry shows that most cells have five or six neighbors. This is also the conclusion in Farhadifar et al. (2007) in both simulations and experiments. The minimum of the potential in Farhadifar et al. (2007), Staple et al. (2010) is obtained in a hexagonal lattice in numerical experiments. In observations of 2D cell patterns in plants in Lewis (1943), it is found that the average number of edges of a cell is six with a hexagonal shape.

A regular polyhedron in 3D with *J* faces or neighbors satisfies

36 $$\begin{aligned} V_i=\sum _j\frac{1}{3}a_{ij}r_i= \frac{1}{3}a_{i}r_i,\; \frac{V_i}{a_{ij}}=\frac{1}{3}Jr_i. \end{aligned}$$

The quotient between $r_i$ and $V^{1/3}$ is written $\xi _3(J)$. Then it follows from (36) that

37 $$\begin{aligned} r_i=\xi _3(J)V_i^{1/3},\; \frac{V_i}{a_{ij}}=\frac{1}{3}J\xi _3(J)V_i^{1/3},\; a_i=\frac{3}{\xi _3(J)}V_i^{2/3}. \end{aligned}$$

We obtain a sphere when $J\rightarrow \infty$. The $\xi _3$-values can be calculated for the five Platonic solids (Weisstein 2003). Four of them are tabulated in Table 2. As in Table 1, the value of $\xi _3$ increases slowly when *J* increases from 6 to $\infty$. The highest density packing of spherical cells is achieved by $J=12$ and the kissing number for a sphere is 12 (Conway and Sloane 1993). Therefore, the icosahedron with $J=20$ is excluded.

**Table 2:** The factor $\xi _3$ in the relation $r=\xi _3(J) V^{1/3}$ in regular polyhedra

J	3D name	$\xi _3$
4	Tetrahedron	$\frac{1}{2\cdot 3^{1/6}}= 0.4163$
6	Cube	0.5000
8	Octahedron	$\frac{1}{2^{2/3}\cdot 3^{1/6}}= 0.5246$
12	Dodecahedron	$\frac{4^{1/3}\sqrt{250+110\sqrt{5}}}{20(15+7\sqrt{5})^{1/3}}= 0.5648$
$\infty$	Sphere	$\left( \frac{3}{4\pi }\right) ^{1/3}= 0.6204$

The formulas in dimension $d,\, d=1,2,3,$ in (33), (35), and (37) are summarized by

38 $$\begin{aligned} r_i=\xi _d(J)V_i^{1/d},\; \frac{V_i}{a_{ij}}=\frac{1}{d}J\xi _d(J)V_i^{1/d},\; a_i=\frac{d}{\xi _d(J)}V_i^{(d-1)/d}. \end{aligned}$$

### Pressure in center and vertex based models

The geometric relations for the cells from Sect. 3.4 are introduced in the pressure formulas from Sects. 3.2 and 3.3.

#### Center based models

Insert $r_{ij}=r_i+r_j$ and $V_i/a_{ij}$ from (38) into the pressure formula for CBM (29) to arrive at

39 $$\begin{aligned} p_{ij}=-\frac{J\xi _d(J)}{d\mu }V_i^{1/d}g(\xi _d(J)(V_i^{1/d}+V_j^{1/d})) \end{aligned}$$

for the pressure between cell *i* and *j*. It can be interpreted as a discretization of

40 $$\begin{aligned} p=-\frac{J\xi _d(J)}{d\mu }V^{1/d}g(2\xi _d(J)V^{1/d}) \end{aligned}$$

at a point $\frac{1}{2}({\mathbf {x}}_i+{\mathbf {x}}_j)$. In *d* dimensions, the pressure formulas for $p_d$ are

41 $$\begin{aligned} p_1=-\frac{1}{\mu }Vg(V),\; p_2=-\frac{1.31}{\mu }V^{1/2}g(1.05V^{1/2}),\; p_3=-\frac{1.83}{\mu }V^{1/3}g(1.1V^{1/3}),\nonumber \\ \end{aligned}$$

with the number of neighbors $J=2, 5, 10,$ for $d=1, 2, 3$. Let *g* be as in (7) and let $s=2\xi _d V_0^{1/d}$. If $V<V_0$ then *p* is positive and decreases when *V* increases. This is the usual behavior in fluids: the pressure decreases when the density ($\sim 1/V$) decreases.

#### Vertex based models

With the VBM force in (15) in 2D, *f* in (31) can be written as a sum

42 $$\begin{aligned} f(V_i,a_j,a_k)=f_V(V_i)+f_a(a_j)+f_a(a_k). \end{aligned}$$

By the geometric relations in 2D we have

43 $$\begin{aligned} -\mu \frac{p_\beta -p_\alpha }{r_{\alpha \beta }}=f_V(V_k)-f_V(V_l)-2\cos (2\pi /J)(f_a(a_k)-f_a(a_l)). \end{aligned}$$

Use (38) with $d=2$ and $r_{\alpha \beta }=a_j/J$ and identify $p_\alpha$ and $p_\beta$ in (43) with

44 $$\begin{aligned} \begin{array}{rl} p_\alpha &{}\displaystyle {=\frac{2}{J\xi _2(J)\mu }V^{1/2}\left( f_V(V_k)-2\cos (2\pi /J)f_a(2V_k^{1/2}/\xi _2(J))\right) ,}\\ p_\beta &{}\displaystyle {=\frac{2}{J\xi _2(J)\mu }V^{1/2}\left( f_V(V_l)-2\cos (2\pi /J)f_a(2V_l^{1/2}/\xi _2(J))\right) } \end{array} \end{aligned}$$

to satisfy the equality. These are interpreted as discrete versions of the pressure in

45 $$\begin{aligned} p=\frac{2}{J\xi _2(J)\mu }V^{1/2}\left( f_V(V)-2\cos (2\pi /J)f_a(2V^{1/2}/\xi _2(J))\right) . \end{aligned}$$

Thus, the pressure corresponding to the Weliky-Oster model in (15) is

46 $$\begin{aligned} p=\frac{2}{J\xi _2(J)\mu }V^{1/2}\left( \frac{\varsigma }{V}-\frac{4\kappa \cos (2\pi /J)}{\xi _2}V^{1/2}\right) . \end{aligned}$$

In summary, we have derived macroscopic constitutive laws for the pressure from the microscopic CBM and VBM in (40) and (46) by comparing a discretization of a pressure gradient with the microscopic forces. Only the cell area or volume *V* is available on the macro level but the CBM and VBM forces depend on more details of the cell geometry such as the perimeter *a* and the distance *r*. The missing information is obtained in (38) by assuming that the cells are regular polygons or polyhedra.

### One dimension

A comparison between the CBM and the VBM for small perturbations in 1D shows how the parameters in the methods are related. A perturbation $\delta V_i$ from the equilibrium $r=s$ in CBM and $V=V_{0}$ in VBM is introduced in the force formulas in Sects. 2.2 and 2.3. The notation is as in Fig. 2.

The CBM velocity of cell *i* according to (28) is

47 $$\begin{aligned} v_i=g(r_i+r_j)\hat{{\mathbf {r}}}_{ij}+g(r_i+r_k)\hat{{\mathbf {r}}}_{ik}. \end{aligned}$$

Perturb $r_i, r_j,$ and $r_k$ about $r_{ij0}=s$. Then the change in velocity is

48 $$\begin{aligned} \delta v_i=g'(s)\delta r_{ij}\hat{{\mathbf {r}}}_{ij}+g'(s)\delta r_{ik}\hat{{\mathbf {r}}}_{ik}=g'(s)(\delta r_{ij}-\delta r_{ik})=g'(s)(\delta r_j-\delta r_k). \end{aligned}$$

The VBM due to Weliky-Oster in (15) depends on the perimeter *a*. It has no immediate interpretation in 1D and is ignored here. The velocity at $x_\alpha$ is

49 $$\begin{aligned} v_\alpha =\varsigma \left( \frac{1}{V_i}-\frac{1}{V_j}\right) =\varsigma (\rho _i-\rho _j). \end{aligned}$$

Perturb the cell lengths as in (48)

50 $$\begin{aligned} \delta v_\alpha =\varsigma \left( \frac{\delta V_j}{V_0^2}-\frac{\delta V_i}{V_0^2}\right) ,\; \delta v_\gamma =\varsigma \left( \frac{\delta V_i}{V_0^2}-\frac{\delta V_k}{V_0^2}\right) , \end{aligned}$$

to obtain

51 $$\begin{aligned} \delta v_i=\frac{1}{2}(\delta v_\alpha +\delta v_\gamma )=\frac{\varsigma }{2V_0^2}\left( \delta V_j-\delta V_k\right) =\frac{\varsigma }{V_0^2}\left( \delta r_j-\delta r_k\right) . \end{aligned}$$

Comparing $\delta v_i$ in (48) and (51) yields $g'(s)=\varsigma /V_0^2$. With *g*(*r*) in (7), $g'(s)=\mu =\varsigma /V_0^2$.

## Finite volume method for the PDE

The equation for conservation of mass (22) is advanced in time by time stepping with the Euler forward method and a FVM discretization in space on an arbitrary mesh as in (24) in this section. A finite difference method or a finite element method would be a possible alternative for the spatial discretization. The pressure in Darcy’s law (25) is defined by the CBM or the VBM on the micro level as in Sect. 3.5.

The conservation law for transport of density is a nonlinear parabolic PDE for $\rho$ in stationary Euler coordinates in (26). Insert $p(\rho )$ into the right hand side of (26)

52 $$\begin{aligned} \nabla \cdot (\rho \nabla p)=\nabla \cdot (\rho \frac{\partial p}{\partial \rho }\nabla \rho ). \end{aligned}$$

The expression $\rho \frac{\partial p}{\partial \rho }$ is a diffusion coefficient in (26). This is observed by Murray et al. (2012) and the coefficient is derived in 1D in a coordinate system different from ours. For $\rho$ to remain stable in the diffusion equation, we require $\frac{\partial p}{\partial \rho }>0$. The diffusion coefficient for the CBM in (40) is with $\rho =1/V$

53 $$\begin{aligned} \frac{\partial p}{\partial \rho }=\frac{J\xi _d(J)}{d^2\mu }\rho ^{-(d+1)/d}(g(2\xi _d\rho ^{-1/d})+2\xi _d\rho ^{-1/d}g'(2\xi _d\rho ^{-1/d})). \end{aligned}$$

In particular the coefficient in 1D is

54 $$\begin{aligned} \frac{\partial p}{\partial \rho }=\frac{1}{\mu \rho ^2}\left( g(1/\rho )+\frac{g'(1/\rho )}{\rho }\right) . \end{aligned}$$

The diffusion coefficient is negative if $\rho$ is such that $g+2\xi _d\rho ^{-1/d}g'<0$ in (53). This is possible e.g. when $2\xi _d\rho ^{-1/d}$ is close to $r_A$ in (7) since $g>0$ but small and $g'<0$. A continuous *g* in (6) with $g>0$ for $s<r<r_A$ and $g=0$ for $r>r_A$ necessarily has this property.

The diffusion coefficient for the VBM in (46) is

55 $$\begin{aligned} \frac{\partial p}{\partial \rho }=\frac{2}{J\xi _2(J)\mu }\left( \frac{\varsigma }{2\rho ^{1/2}}+\frac{4\kappa \cos (2\pi /J)}{\xi _2(J)\rho ^2}\right) . \end{aligned}$$

The coefficient is positive when $J\ge 4$ with stable solutions of (26).

Compressible fluids are modelled by constitutive laws for the relation between *p* and $\rho$ or *V* similar to (40) and (46) (Anderson 1990). A perfect gas satisfies

56 $$\begin{aligned} p=\rho R T \end{aligned}$$

where *R* is the specific gas constant and *T* is the temperature. The constitutive law for an isentropic gas such as air ($\gamma =1.4$) is

57 $$\begin{aligned} \frac{p}{p_0}=\left( \frac{\rho }{\rho _0}\right) ^{\gamma }. \end{aligned}$$

The assumption in Chaplain et al. (2020) is that $p(\rho )=K_\gamma \rho ^\gamma ,\, K_\gamma>0,\, \gamma >1$, as in (57).

The space derivatives in (26) are discretized by a finite volume method on an arbitrary stationary mesh with element areas (or volumes) $\omega _i, \,\omega _j$. The separating line (or area) between $\omega _i$ and $\omega _j$ is denoted by $\sigma _{ij}$ and the distance between the element centers is $\ell _{ij}$. Then the equation for $\rho _i$ in element *i* is

58 $$\begin{aligned} \begin{array}{ll} \displaystyle {\frac{\partial \rho _i}{\partial t}}=\displaystyle {\frac{\mu }{\omega _i}\sum _{j\in \mathcal {J}_i}\rho _{ij}{\mathbf {n}}_{ij}\cdot (\nabla p)_{ij}\sigma _{ij} =\frac{\mu }{\omega _i}\sum _{j\in \mathcal {J}_i}\frac{1}{2}(\rho _{i}+\rho _{j})\frac{p_{j}-p_{i}}{\ell _{ij}}\sigma _{ij}}. \end{array} \end{aligned}$$

The equation (58) is integrated in time from $t^n$ to $t^{n+1}$ by the forward Euler method with the time step $\varDelta t$ resulting in

59 $$\begin{aligned} \rho _i^{n+1}=\rho _i^n+\frac{\mu \varDelta t}{\omega _i}\sum _{j\in \mathcal {J}_i}\frac{1}{2}(\rho _{i}+\rho _{j})\frac{p_{j}-p_{i}}{\ell _{ij}}\sigma _{ij}. \end{aligned}$$

The right hand side is evaluated at $t^n$. On a Cartesian equidistant spatial grid with grid size $\varDelta x$, we have $\sigma _{ij}=\varDelta x^{d-1}, \ell _{ij}=\varDelta x$, and $\omega _i=\varDelta x^d$.

Multiply (59) by $\omega _i$ and sum over all *M* elements in the mesh

60 $$\begin{aligned} \sum _{i=1}^M\omega _i\rho _i^{n+1}=\sum _{i=1}^M\omega _i\rho _i^n +\mu \varDelta t\sum _{i=1}^M\sum _{j\in \mathcal {J}_i}\frac{1}{2}(\rho _{i}+\rho _{j})\frac{p_{j}-p_{i}}{\ell _{ij}}\sigma _{ij}=\sum _{i=1}^M\omega _i\rho _i^n.\nonumber \\ \end{aligned}$$

The double sum over *i* and *j* vanishes since each term in the interior of the domain appears twice with opposite sign and a term on the boundary is zero due to the discretized Neumann boundary condition $\rho _i=\rho _j$ implying $p_i=p(\rho _i)=p(\rho _j)=p_j$. Therefore, the total mass $\sum _i\omega _i\rho _i$ is conserved also in the discretization, cf. $\rho$ in the PDE in (22).

The pressure at *i* and *j* in (59) is approximated by the CBM in dD in (40)

61 $$\begin{aligned} p_k=-\frac{J\xi _d(J)}{d\mu }V_k^{1/d}g(2\xi _d(J) V_k^{1/d}),\; k=i,j, \end{aligned}$$

with the specific volume $V_k$ resulting in

62 $$\begin{aligned} \begin{array}{rl} \displaystyle {\rho _i^{n+1}=\rho _i^n-\frac{J\xi _d\varDelta t}{d\omega _i}\sum _{j\in \mathcal {J}_i}}&{}\displaystyle {\frac{\sigma _{ij}}{2\ell _{ij}}(\rho _{i}+\rho _{j})}\\ &{}\cdot (V_j^{1/d}g(2\xi _d(J) V_j^{1/d})-V_i^{1/d}g(2\xi _d(J) V_i^{1/d})). \end{array} \end{aligned}$$

The constant *J* in (62) is chosen to be fixed with different values in 2D and 3D. It is 6 in the numerical experiments in 2D in the next section assuming that the cell environment is dense and slowly changing. The PDE model is not suitable in rapidly changing domains. There the distance *r* may not depend on *V* as smoothly as in (38) where a change in $V_i$ will change $r_i$ equally in all directions from the cell center.

The approximation of *p* at ${\mathbf {x}}_k$ with the Weliky-Oster model (46) in 2D is

63 $$\begin{aligned} p_k=\frac{2}{J\xi _2(J)\mu }V_k^{1/2}\left( \frac{\zeta }{V_k}-\frac{4\cos (2\pi /J)\kappa }{\xi _2(J)}V_k^{1/2}\right) ,\; k=i,j. \end{aligned}$$

This *p* is inserted into (59) to advance the cell system in time with the VBM.

The PDE is discretized on an equidistant grid in 1D with grid size $\varDelta x$ with CBM forces (40) and indices as in Fig. 2. Let $\rho _{ij}=\frac{1}{2}(\rho _{i}+\rho _{j})$ and update $\rho$ in time by

64 $$\begin{aligned} \begin{array}{rl} \rho _i^{n+1}&{}=\displaystyle {\rho _i^n-\frac{\varDelta t}{\varDelta x^2}\sum _{j\in \mathcal {J}_i}\rho _{ij}(V_jg(V_j)-V_ig(V_i))}\\ &{}=\displaystyle {\rho _i^n-\frac{\varDelta t}{\varDelta x^2}\left( \rho _{ij}(V_jg(V_j)-V_ig(V_i))+\rho _{ki}(V_kg(V_k)-V_ig(V_i))\right) }. \end{array} \end{aligned}$$

Apply FVM to (22) for a comparison of the PDE in 1D with the discrete CBM model. In CBM, $v_{ij}=g(V_j)-g(V_i)$ and (22) is discretized by

65 $$\begin{aligned} \begin{array}{rl} \rho _i^{n+1}&{}=\displaystyle {\rho _i^n-\frac{\varDelta t}{V_i}(\rho _{ij}v_{ij}-\rho _{ki}v_{ki})}\\ &{}=\displaystyle {\rho _i^n-\frac{\varDelta t}{V_i^2}\left( \rho _{ij}V_i(g(V_j)-g(V_i))+\rho _{ki}V_i(g(V_k)-g(V_i))\right) }. \end{array} \end{aligned}$$

The difference between the PDE solution in (64) and the CBM solution in (65) is in the evaluation of the pressure and the variable cell size in (65).

The pressure in the Weliky-Oster model in 1D is derived from (46). The constant $2/(J\xi _2)\rightarrow 1/(2\xi _1)=1$ in 1D and $f_a=0$. Multiplying the force function by *V* results in a constant *p*

$$\begin{aligned} p=\frac{1}{\mu } Vf(V)=\frac{\varsigma }{\mu }. \end{aligned}$$

This is avoided by letting a discrete pressure $p_j$ at $x_j$ to update element *i* be

66 $$\begin{aligned} p_j=\frac{1}{\mu }V_if(V_j). \end{aligned}$$

Then the integration of $\rho ^n_i$ is achieved by

67 $$\begin{aligned} \rho _i^{n+1}=\rho _i^n+\frac{\varDelta t}{\varDelta x^2}\left( \rho _{ij}V_i(f(V_j)-f(V_i))+\rho _{ki}V_i(f(V_k)-f(V_i))\right) , \end{aligned}$$

cf. (64) and (65).

With a general macroscopic definition of the pressure as in (40), it is possible to integrate the vertex coordinates ${\mathbf {x}}_\alpha$ in (13) with the CBM force by taking

68 $$\begin{aligned} f(V)=-\left( \frac{J\xi _2(J)}{2}\right) ^2g(2\xi _2(J)V^{1/2}). \end{aligned}$$

The macroscopic pressure in the PDE is the same with this *f*. In the same manner, *p* in (45) can be used to advance the center coordinates in (10) with a *g* derived from (68).

## Numerical results

The discrete methods and their PDE approximations in Sect. 3 are compared in this section in numerical experiments in 1D and 2D. The methods are implemented in Matlab in a straightforward manner using their voronoin for the Voronoi tesselation without any attempts to optimize the efficiency of the code. The parameters in the methods are displayed in Table 3.

**Table 3:** The parameters in the CBM in (7) and the VBM in (14)

Method	Parameter	Value	Method	Parameter	Value
CBM	*s*	1	VBM	$\varsigma$	1, 5, 50
CBM	$\mu$	50	VBM	$\kappa$	1, 2, 5
CBM	*c*	10			
CBM	$r_A$	1.5			

The distance *r* between two cell centers with no force between them is denoted by *s* in the CBM. The force is scaled by $\mu$. The exponential decay of the adhesion force is given by *c* and the force vanishes when $r>r_A$. In the VBM, $\varsigma$ and $\kappa$ are the scalings of the area and the perimeter dependent force components, respectively

### One dimension

The density of the particles is computed in 1D using the CBM and VBM forces in the micro model and the corresponding macro level PDEs in a comparison of the methods in Figs. 4, 5, 6, 7. The parameters in the methods are found in Table 3. The relation between the parameters $\varsigma$ and $\kappa$ in the VBM is important but not the scaling of them. It follows from (10) and (13) that a different parameter scale of $\mu , \varsigma ,$ and $\kappa$ will only change the time scale of the evolution of the system.

A system consisting of *N* particles is simulated for $t\ge 0$ in an interval $\mathcal {A}$ in space. The spatial cells or intervals are computed by a Voronoi tesselation of the particle system as in (32). Then the cell density in the interval is given by $\rho _i=1/V_i$. The original positions of the cells at $t=0$ are such that $\rho$ varies smoothly. The initial density distribution is interpolated to a grid with constant step size $\varDelta x$ for use as initial conditions to the solution of the PDE discretized by FVM in the interval $\mathcal {B}$. The cells at the boundaries of $\mathcal {A}$ are fixed and $\rho$ satisfies Neumann conditions at the boundaries of $\mathcal {B}$. These boundary conditions guarantee that mass is conserved in $\mathcal {A}$ and $\mathcal {B}$. A stationary or equilibrium solution for the discrete model and the continuum model have a constant density with equal cell size.

**Fig. 3:**
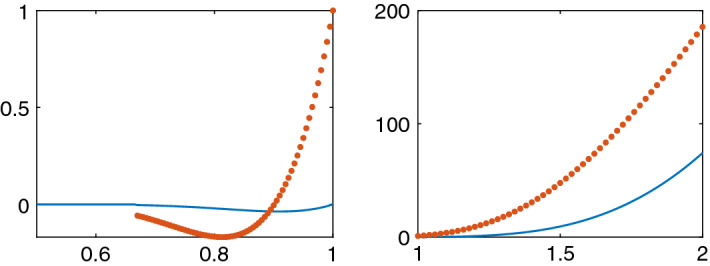
The pressure *p* (solid blue) and the diffusion coefficient $\rho \frac{\partial p}{\partial \rho }$ (dotted red) in the CBM in 1D in the $\rho$-intervals [0.5, 1] (left) and [1, 2] (right). The parameters in the CBM are as in Table 3

**Fig. 4:**
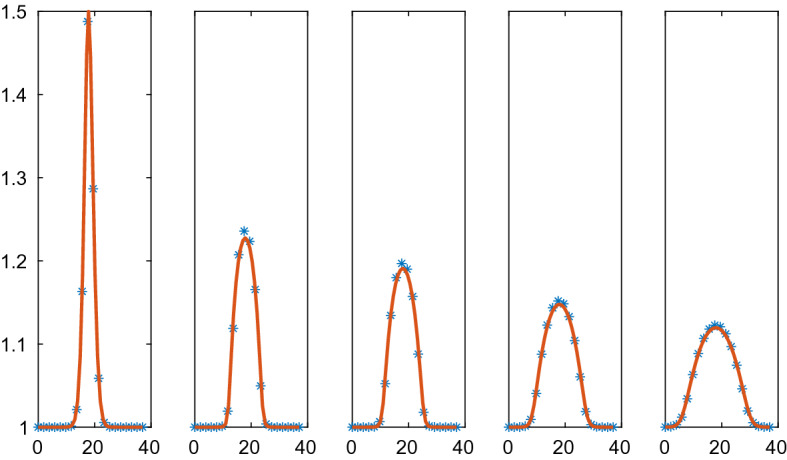
Comparison of the density in the CBM and the PDE solutions in 1D in $\mathcal {A}=\mathcal {B}=[0, 37]$ at different times *t*. The CBM and PDE solutions are marked by a solid line (red) and $*$ (blue), respectively. In the columns from left to right: $t=0, 0.01, 0.02, 0.05, 0.1$. The timestep $\varDelta t=1.25\cdot 10^{-4}$, $N=40$, and the grid size is $\varDelta x=1.95$

**Fig. 5:**
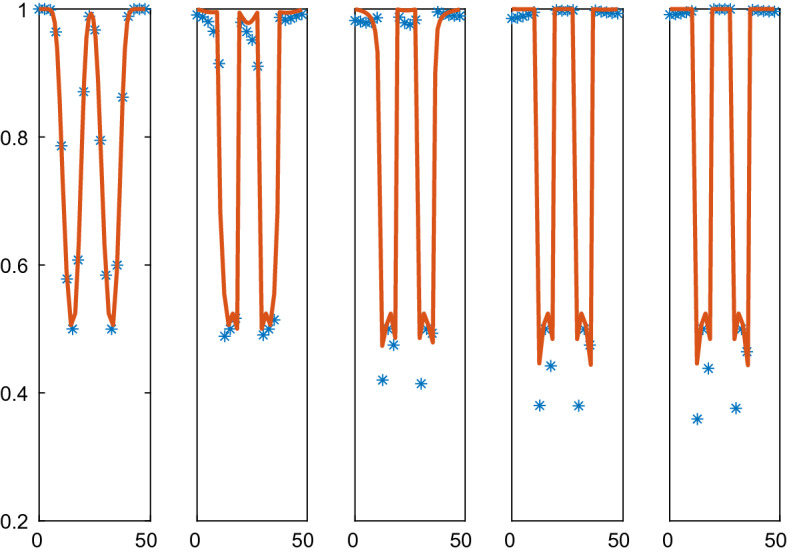
Comparison of the density in the CBM and the PDE solutions in 1D in $\mathcal {A}=\mathcal {B}=[0, 47]$ at different times *t*. The CBM and PDE solutions are marked by a solid line (red) and $*$ (blue), respectively. In the columns from left to right: $t=0, 0.5, 1.0, 2.0, 3.0$. The timestep $\varDelta t=10^{-3}$, $N=40$, and the grid size is $\varDelta x=2.50$

The repulsion and adhesion between the cells is modelled by the CBM in (7) in the first example. The behavior of $p(\rho )$ based on CBM with the parameters in Table 3 is depicted in Fig. 3. The particle simulation with $N=40$ and the solution of the PDE in (64) are found in Figs. 4 and 5. The initial data have a peak compared to the equilibrium density in Fig. 4 and the density is lower in parts of the domain in Fig. 5. When $\rho >1$ in Fig. 4 then there is a repulsive force between the cells in the discrete model and $\partial p/\partial \rho >0$ in (54) in the PDE model (to the right in Fig. 3). The solution diffuses toward the constant state. An adhesive force acts between the cells when $1/r_A=0.667<\rho <1$ and the force disappears when $\rho <0.667$. The diffusion in the PDE is negative and unstable when $\rho <0.901$ and vanishes when $\rho <0.667$ (to the left in Fig. 3). For small perturbations about $r_{ij}=s$, the effect is given by $g'(s)$ in (48) which is equal to $\mu$ with the force in (7).

In Fig. 5, the solutions approach a steady state with two $\rho$-levels: one low with separated cells and one high with cells touching each other. Because of the instability in the PDE for low $\rho$, some of the $\rho$ values in the solution decrease as time increases. The solutions are close to steady state at $t=3$ and the high density solutions agree between the CBM and the PDE. The instability in the PDE causes oscillations in the numerical solution for the low density values. This phenomenon is not ameliorated by refining the mesh or reducing the timestep. Since the diffusion vanishes for low density values, the oscillations remain in the stationary solution.

**Fig. 6:**
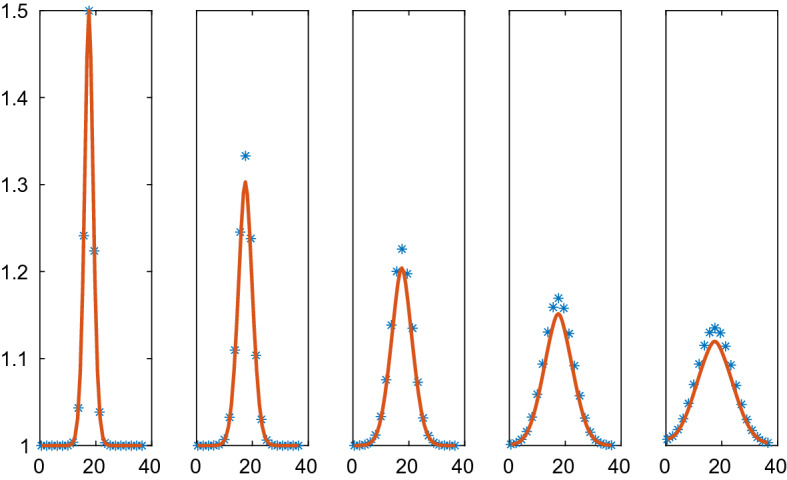
Comparison of the density in the VBM and the PDE solutions in 1D in $\mathcal {A}=\mathcal {B}=[0.5, 36.5]$ at different times *t*. The VBM and PDE solutions are marked by a solid line (red) and $*$ (blue), respectively. In the columns from left to right: $t=0, 0.04, 0.12, 0.24, 0.4$. The timestep $\varDelta t=4\cdot 10^{-4}$, $N=39$, and the grid size is $\varDelta x=1.90$

**Fig. 7:**
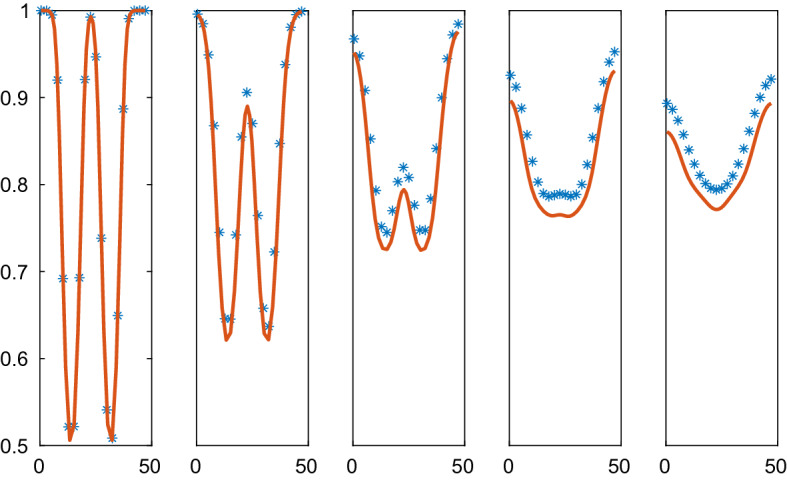
Comparison of the density in the VBM and the PDE solutions in 1D in $\mathcal {A}=\mathcal {B}=[0.5, 47]$ at different times *t*. The VBM and PDE solutions are marked by a solid line (red) and $*$ (blue), respectively. In the columns from left to right: $t=0, 0.1, 0.3, 0.6, 1.0$. The timestep $\varDelta t=10^{-3}$, $N=39$, and the grid size is $\varDelta x=2.45$

The Weliky-Oster VBM in (14) is compared with the corresponding PDE in Figs. 6 and 7. The PDE is stable with a positive diffusion coefficient. The initial data and the boundary conditions are the same as in the CBM example above and the force coefficient is $\varsigma =50$. The vertices of 39 cells are advected in the micro model and the macro model has 20 grid points. As observed in Sect. 4, there is no directly corresponding continuous pressure in 1D for the VBM but a discretization of the PDE is still possible with the pressure (66) in (67) which yields a fair result. The PDE solutions diffuse toward a constant steady state as time progresses which is different from the steady state with CBM in Fig. 5. The discrete and the PDE solutions are close except for the peak in $\rho$. Peaks cannot be represented very well in a PDE discretization on a coarse mesh.

### Two dimensions

**Fig. 8:**
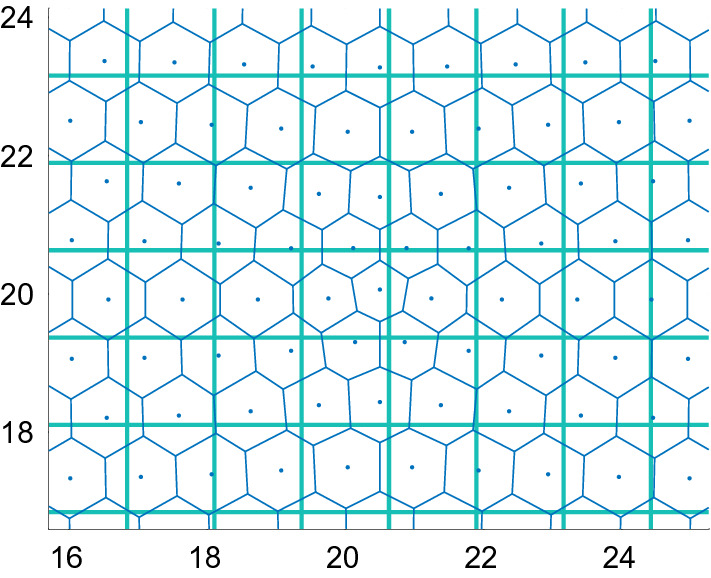
An example of a Voronoi tesselation (blue) of a collection of cells with cell centers marked by $\cdot$. The Cartesian grid (green) is overlaid

The density of the particles is computed in 2D in this section using the CBM and VBM forces and the corresponding PDEs in a comparison of the methods. The parameters in Table 3 are used in the methods. A system consisting of 1777 particles in $\mathcal {A}=[0,40]\times [0,40]$ is simulated in a time interval with $t\ge 0$. The density of the particle system is computed by a Voronoi tesselation. The area $V_i$ of the Voronoi cell is first determined and then the density by $\rho _i=1/V_i$. The original positions of the cells are located in a hexagonal pattern. It is disturbed in the center of the domain such that the density varies radially from there. An example is found in Fig. 8 with $\rho \in [1.11, 1.97]$ in the central parts of the figure and $\rho =1.15$ in the outer parts. The cells will be close to hexagons also in the steady state. Therefore, *J* is chosen to be 6 in $\xi _2(J)$. The PDE is discretized by FVM on a Cartesian grid with square elements in $\mathcal {B}=[8,32]\times [8,32]$ as in (59) with the grid sizes $\varDelta x=\varDelta y=1.263$, see Fig. 8. The initial $\rho$ in the PDE solution at $t=0$ is interpolated to the grid from the initial CBM or VBM solution. The solutions of the discrete system and the PDEs are compared along a line with a constant *y* value through the center of the domain along the *x* axis.

**Fig. 9:**
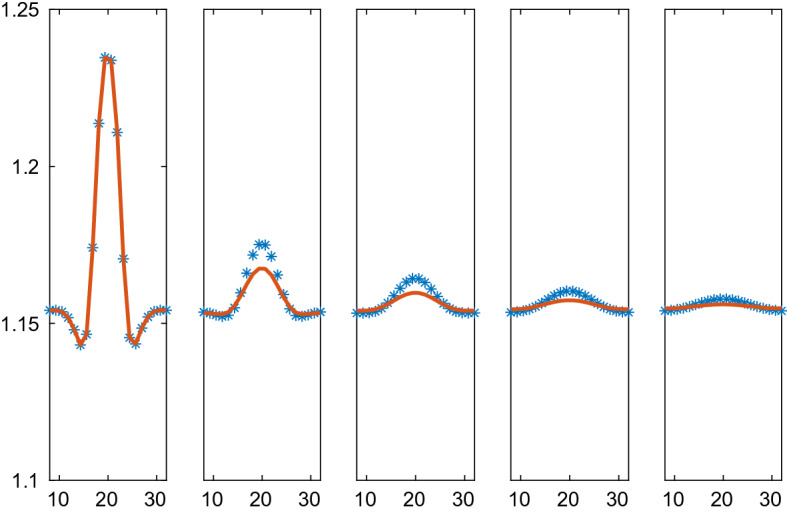
Comparison of the density in the CBM and the PDE solutions in 2D on the line through the center of the solution at different times *t*. The CBM and PDE solutions are marked by a solid line (red) and $*$ (blue), respectively. In the columns from left to right: $t=0, 0.1, 0.2, 0.3, 0.45$

The system with CBM forces is simulated for $t\in [0,0.45]$ with the timesteps $\varDelta t=2.5\cdot 10^{-4}$. The CBM solution and the PDE solution of (62) are compared at five time points in Fig. 9. The PDE solution decays at a slower rate than the CBM solution. The explanation is that *r* in the force formula varies more rapidly in a neighborhood of large variations in $\rho$ than *V* does in the PDE approximation $r=\xi _2V^{1/2}$ thus inducing weaker forces and slower dissipation in the PDE solution. The cells at the center of the domain are less regular than assumed to obtain $\xi _2$. Refining the PDE mesh does not change the result very much. The steady state solution is a constant density and the boundary conditions are such that the total mass is conserved. Close to the steady state at $t=0.45$, the solutions almost overlap on the scale of the figure. When $t\rightarrow \infty$, $\rho$ will approach the average at $t=0.45$. It is 1.1558 for the micro solution and 1.1554 for the macro solution.

**Fig. 10:**
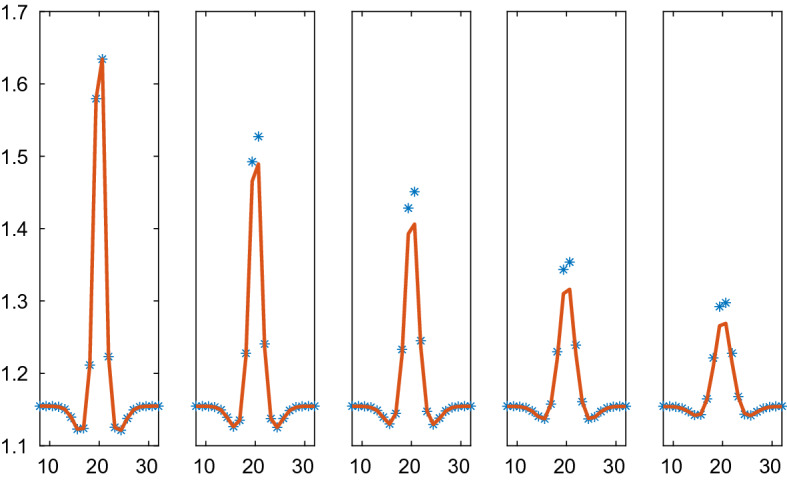
Comparison of the density in the VBM and the PDE solutions with Weliky and Oster forces in 2D on the line through the center of the solution at different times *t* with parameters $\varsigma =1, \kappa =1$. The VBM and PDE solutions are marked by a solid line (red) and $*$ (blue), respectively. In the columns from left to right: $t=0, 0.1, 0.2, 0.4, 0.6$

**Fig. 11:**
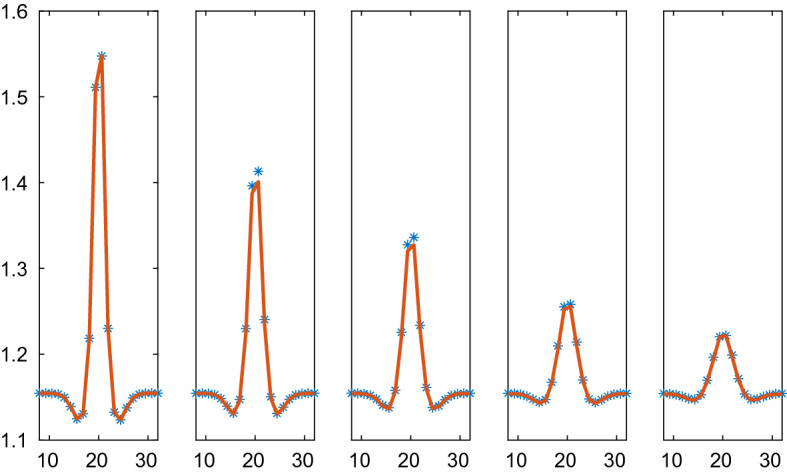
Comparison of the density in the VBM and the PDE solutions with Weliky and Oster forces in 2D on the line through the center of the solution at different times *t* with parameters $\varsigma =1, \kappa =2$. The VBM and PDE solutions are marked by a solid line (red) and $*$ (blue), respectively. In the columns from left to right: $t=0, 0.1, 0.2, 0.4, 0.6$

**Fig. 12:**
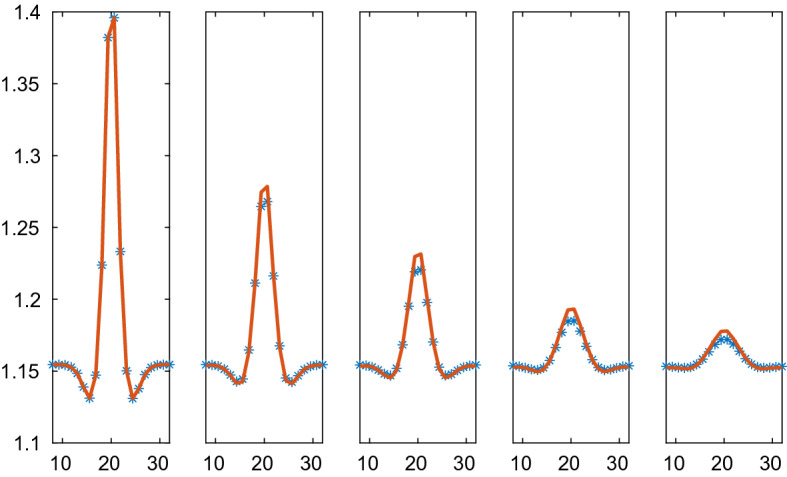
Comparison of the density in the VBM and the PDE solutions with Weliky and Oster forces in 2D on the line through the center of the solution at different times *t* with parameters $\varsigma =1, \kappa =5$. The VBM and PDE solutions are marked by a solid line (red) and $*$ (blue), respectively. In the columns from left to right: $t=0, 0.1, 0.2, 0.4, 0.6$

**Fig. 13:**
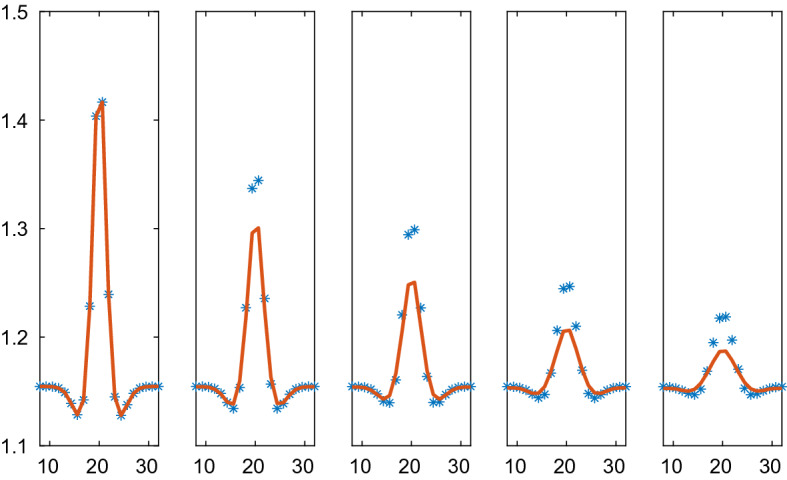
Comparison of the density in the VBM and the PDE solutions with Weliky and Oster forces in 2D on the line through the center of the solution at different times *t* with parameters $\varsigma =5, \kappa =1$. The VBM and PDE solutions are marked by a solid line (red) and $*$ (blue), respectively. In the columns from left to right: $t=0, 0.1, 0.2, 0.4, 0.6$

The solutions with the VBM forces due to Weliky and Oster are compared at five time points in the interval [0, 0.6] in Figs. 10, 11, 12, and 13 for different parameters $\varsigma$ and $\kappa$. The timestep is $\varDelta t=10^{-3}$. The difference between the particle simulation and the PDE solution of (59) with the pressure (63) is largest at the peak of the density. There the approximations of a length scale *r* by $\xi _2V^{1/2}$ and the perimeter *a* by $2V^{1/2}/\xi _2$ are the least accurate to determine a pressure for the PDE.

In the figures, the solutions computed by the CBM and the VBM and the PDE approximations are compared. The computing time to obtain the solutions is at least an order of magnitude faster with the PDEs. For example, take the simulation in Fig. 10. Initialization of data structures necessary for a VBM simulation required 84.9 s on a standard processor. The VBM simulation itself with 800 time steps took 217.2 s. The PDE solution with 800 time steps was finished in 10.5 s. These numbers give a hint of the relations between the computational work for the micro and macro levels of simulation.

## Conclusions

The CBM and the VBM are models on a micro level for the mechanical forces between neighboring biological cells in time dependent motion. Each cell is treated as an entity whose motion is determined by the forces. PDEs are suitable models on a macro level for the motion of large aggregations of cells. We have derived these PDEs from two established CBM and VBM in Sect. 3 and compared the two levels of modelling in numerical examples in Sect. 5. The advantage with PDEs is that tissues with billions of cells are easier to simulate than the detailed particle models with the same number of cells. A disadvantage with a PDE is that local events such as cell proliferation cannot be well represented.

The PDE on the macro level, corresponding to the CBM and the VBM on the micro level, is nonlinear and parabolic. It is an equation for transport of cell density $\rho =1/V$ where *V* is the area or volume of a cell. The forces in CBM and VBM are transformed to a pressure *p* as a function of *V*, *p*(*V*),  in the PDE and there is a direct translation of the parameters in the microscopic and the macroscopic models. By the relation between the forces and the pressure, a pressure obtained from a CBM force can be used to define a VBM force and vice versa. The microscopic forces depend on the cell perimeter *a* in 2D and the cell radius *r* in 2D and 3D. They are involved in the specification of the macroscopic pressure but are not readily available on the macro level. This is usually the case in a multiscale model that information on the micro level is missing on the macro level. Instead, *a* and *r* are approximated in the PDE assuming that the cell is a regular polygon in 2D and a regular polyhedron in 3D. This approximation is not necessary in 1D. The PDE derived from the CBM is unstable in a density interval because the diffusion coefficient is negative there. The motion of the cells is assumed to obey Darcy’s law, which governs the flow of a fluid through a porous medium. An alternative would be to assume that the cell aggregation behaves as a solid tissue with shear forces such as a tumour described by a nonlinear elastic model (Lowengrub et al. 2010) and then relate a given microscale model to those equations or choose a micro model suitable for those equations.

There are discrepancies between the solutions in 2D computed with the discrete particle models and the PDE approximations in the numerical experiments in Sect. 5. These are explained by the approximations of *a* and *r* in the PDE which are less accurate at peaks in the density distribution where the cell geometry deviates the most from a regular polygon. In 1D, the agreement between the fine micro level and the coarser macro level solutions is good. A possible solution in dimensions higher than one is to derive transport equations for *a* and *r* in the same spirit as for *V* (or $\rho$) and use these *a*, *r*,  and *V* to define *p*. This would be more complicated than it is to arrive at an equation for $\rho$ and would require detailed knowledge on the macro level of the initial cell geometry to provide initial data for *a* and *r*. Another solution is to let the forces between the cells on the micro level depend only on the cell volume *V* to simplify the transfer to the macro level. In this way, the macroscopic model would define the forces in the microscopic model to which additional, detailed features could be added to motivate and refine a cell based approach.

The advantage with a PDE model is that the spatial discretization of it for numerical solution is chosen to resolve the variation in the density without regard to the size of the biological cell. If the cell size is almost constant and the density varies smoothly, then major reductions in computational work are possible by a coarse mesh on the macro level. With a micro model and a macro model one can treat parts of the cell domain by the CBM or VBM where detailed modelling is required and more quiescent parts by the PDE model and glue them together at an interface in a multiscale model as in Kim et al. (2007). The PDE would define conditions at the boundary of an embedded domain with discrete, microscopic modelling. That appears to be possible already with the PDEs in Sect. 5 if the variation in density is low in the PDE domain.

## Declarations

### Code availability

The Matlab code is available from the author upon request.
